# Supplementary figures and images for: UHPLC/MS-Based Untargeted Metabolomics Reveals Metabolic Characteristics of Clinical Strain of Mycoplasma bovis
Source: Microorganisms. 2023 Oct 21;11(10):2602. doi: 10.3390/microorganisms11102602 (PMC10608813; doi:10.3390/microorganisms11102602)

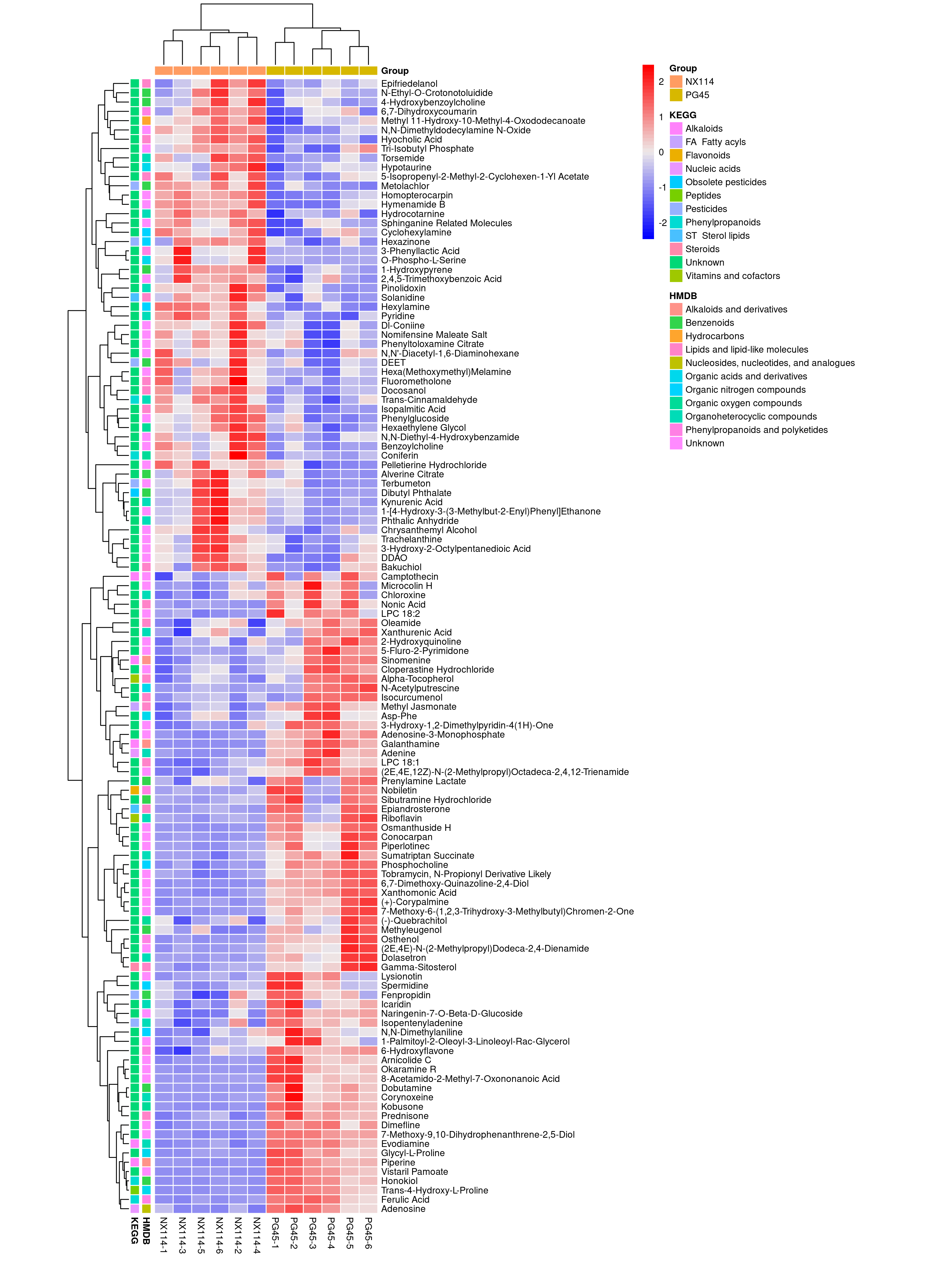

Supplement: Supplementary file 1 [file microorganisms-11-02602-s001.zip › microorganisms-2572227-supplementary/Supplementary material/Figure S1 a.png]

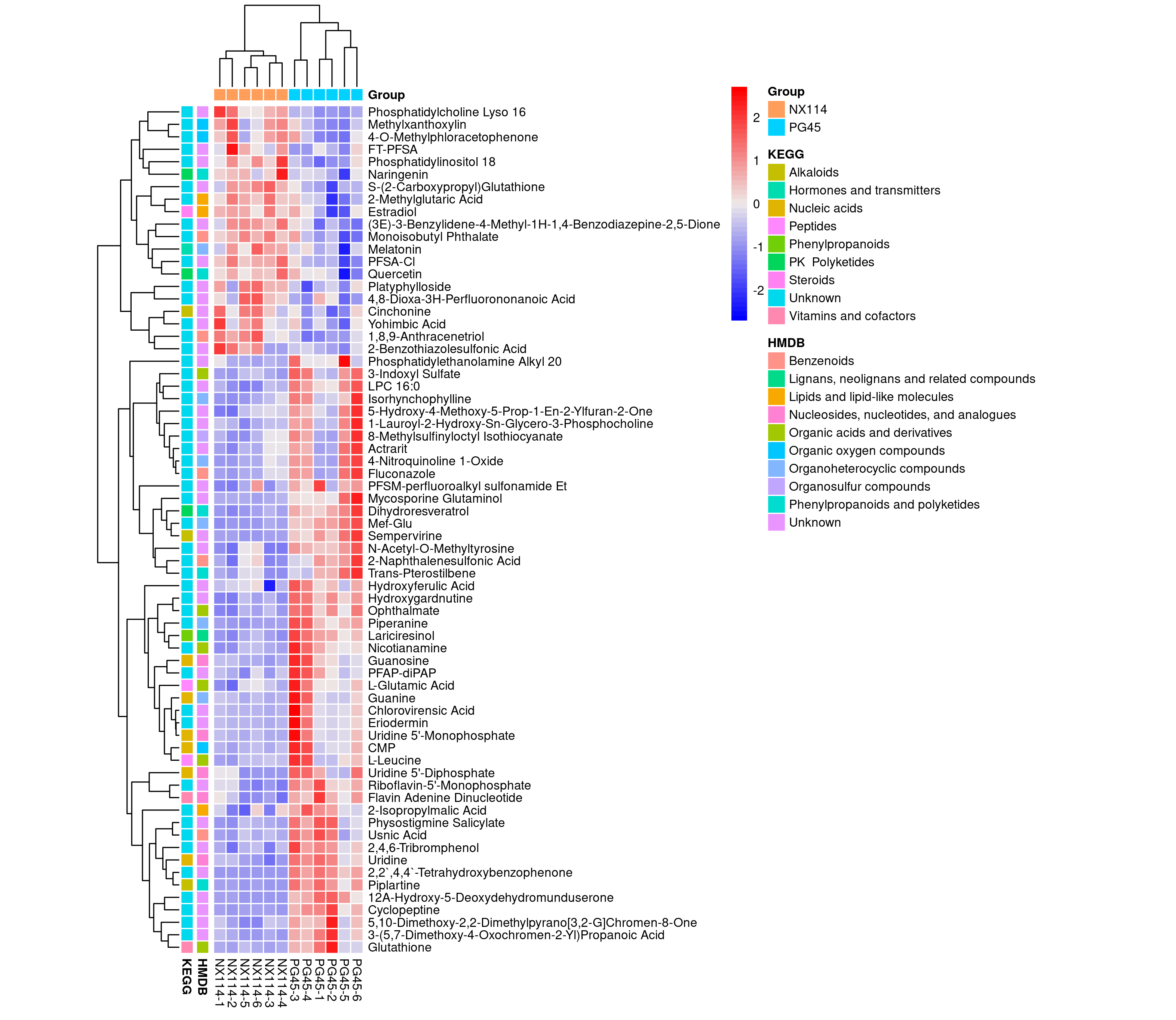

Supplement: Supplementary file 1 [file microorganisms-11-02602-s001.zip › microorganisms-2572227-supplementary/Supplementary material/Figure S1 b.png]
